# Intercalary Resection of the Tibia for Primary Bone Tumors: Are Vascularized Fibula Autografts With or Without Allografts a Durable Reconstruction?

**DOI:** 10.1097/CORR.0000000000003007

**Published:** 2024-03-21

**Authors:** Domenico Andrea Campanacci, Roberto Scanferla, Mariagrazia Marsico, Federico Scolari, Guido Scoccianti, Giovanni Beltrami, Luca Delcroix, Marco Innocenti, Rodolfo Capanna

**Affiliations:** 1 Department of Orthopaedic Oncology and Reconstructive Surgery, Careggi University Hospital, Florence, Italy; 2 Department of Paediatric Orthopaedics, Meyer University Hospital, Florence, Italy; 3 Department of Plastic Surgery, Careggi University Hospital, Florence, Italy; 4 Department of Plastic Surgery, Rizzoli Orthopaedic Institute, Bologna, Italy; 5 Orthopaedic Clinic, Cisanello University Hospital, Pisa, Italy

## Abstract

**Background:**

Reconstruction with vascularized fibula grafts (VFG) after intercalary resection of sarcoma may offer longevity by providing early graft-host union and fracture healing. The ability of the fibula to hypertrophy under mechanical stress, as well as vascularized bone in the area, may also be advantageous, given that soft tissues may be compromised because of resection, chemotherapy, or radiation therapy. VFG with a massive allograft combines the primary mechanical stability of the graft with the biological potential of the vascularized fibula; however, complications and the durability of this combined reconstruction are not well described.

**Questions/purposes:**

(1) What was the proportion of complications after reconstruction with VFG, with or without allografts? (2) What was the functional result after surgical treatment as assessed by the Musculoskeletal Tumor Society (MSTS) score? (3) What was the survivorship of these grafts free from revision and graft removal?

**Methods:**

Between 1988 and 2021, 219 patients were treated at our institution for a primary malignant or aggressive benign bone tumor of the tibia with en bloc resection. Of those, 54% (119 of 219) had proximal tibial tumors with epiphyseal involvement and were treated with either intra-articular resection and reconstruction with an osteoarticular allograft, allograft-prosthesis composite (APC), or modular prosthesis according to age, diagnosis, and preoperative or postoperative radiotherapy. Nine percent (20) of patients had distal tibial tumors that were treated with intra-articular resection and reconstruction with ankle arthrodesis using allogenic or autologous grafts, and 0.5% (1 patient) underwent total tibial resection for extensive tumoral involvement of the tibia and reconstruction with an APC. Thirty-six percent (79) of patients had a metadiaphyseal bone tumor of the tibia and were treated with intercalary joint-sparing resection. We routinely use reconstruction with VFG after intercalary tibial resection for primary malignant or aggressive benign bone tumors in patients with long life expectancy and high functional demands and in whom at least 1 cm of residual bone stock of the proximal or distal epiphysis can be preserved. By contrast, we routinely use intercalary massive allograft reconstruction in short resections or in patients with metastatic disease who do not have long life expectancy. We avoid VFG in patients with tibial bone metastasis, patients older than 70 years, or primary bone tumors in patients who may undergo postoperative radiotherapy; in these patients, we use alternative reconstructive methods such as intercalary prostheses, plate and cement, or intramedullary nailing with cement augmentation. According to the above-mentioned indications, 6% (5 of 79) of patients underwent massive allograft reconstruction because they were young and had intercalary resections shorter than 7 cm or had metastatic disease at diagnosis without long life expectancy, whereas 94% (74) of patients underwent VFG reconstruction. The median age at operation was 16 years (range 5 to 68 years). The diagnosis was high-grade osteosarcoma in 22 patients, Ewing sarcoma in 19, adamantinoma in 16, low-grade osteosarcoma in five, fibrosarcoma in three, malignant fibrous histiocytoma and Grade 2 chondrosarcoma in two, and malignant myoepitelioma, angiosarcoma of bone, malignant peripheral nerve sheath tumor of bone, squamous cell carcinoma secondary to chronic osteomyelitis, and desmoplastic fibroma in one patient each. Median follow-up was 12.3 years (range 2 to 35 years). The median tibial resection length was 15 cm (range 7 to 27 cm), and the median fibular resection length was 18 cm (range 10 to 29 cm). VFG was used with a massive allograft in 55 patients, alone in 12 patients, and combined with allogenic cortical bone struts in seven patients. We used VFG combined with a massive allograft in patients undergoing juxta-articular, joint-sparing resections that left less than 3 cm of residual epiphyseal bone, for intra-epiphyseal resections, or for long intercalary resections wherein the allograft can provide better mechanical stability. In these clinical situations, the combination of a VFG and massive allograft allows more stable fixation and better tendinous reattachment of the patellar tendon. VFG was used with cortical bone struts in distal tibia intercalary resections where the narrow diameter of the allograft did not allow concentric assembling with the fibula. Finally, VFG alone was often used after mid- or distal tibia intercalary resection in patients with critical soft tissue conditions because of previous surgery, in whom the combination with massive allograft would result in a bulkier reconstruction. We ascertained complications and MSTS scores by chart review, and survivorship free from revision and graft removal was calculated using the Kaplan-Meier estimator. In our study, however, the occurrence of death as a competing event was observed in a relatively low proportion of patients, and only occurred after the primary event of interest had already occurred. Considering the nature of our data, we did not consider death after the primary event of interest as a competing event.

**Results:**

In all, 49% (36 of 74) of patients experienced complications and underwent operative treatment. There were 45 complications in 36 patients. There was one instance of footdrop secondary to common peroneal nerve palsy, four wound problems, one acute vein thrombosis of the VFG pedicle and one necrosis of the skin island, two episodes of implant-related pain, 10 nonunions, six fractures, six deep infections, nine local recurrences, one Achilles tendon retraction, one varus deformity of the proximal tibia with postoperative tibial apophysis detachment, one knee osteoarthritis, and one hypometria. The median MSTS score was 30 (range 23 to 30); the MSTS score was assessed only in patients in whom the VFG was retained at the final clinical visit, although if we had considered those who had an amputation, the overall score would be lower. Revision-free survival of the reconstructions was 58% (95% confidence interval 47% to 70%) at 5 years, 52% (95% CI 41% to 65%) at 10 and 15 years, and 49% (95% CI 38% to 63%) at 20 and 30 years. Eight patients underwent VFG removal because of complications, with an overall reconstruction survival of 91% (95% CI 84% to 98%) at 5 years and 89% (95% CI 82% to 97%) at 10 to 30 years.

**Conclusion:**

VFG, alone or combined with an allograft, could be considered in reconstructing a lower extremity after intercalary resections of the tibia for primary bone tumors, and it avoids the use of a large endoprosthesis. However, this procedure was associated with frequent, often severe complications during the first postoperative years and complication-free survival of 58% at 5 years. Nearly 10% of patients ultimately had an amputation. For patients whose reconstruction succeeded, the technique provides a durable reconstruction with good MSTS scores, and we believe it is useful for active patients with long life expectancy. Fractures, frequently observed in the first 5 years postoperatively, might be reduced using long-spanning plate fixation, and that appeared to be the case in our study. Nonbridging fixation can be an option in intraepiphyseal resection when a spanning plate cannot be used or in pediatric patients to enhance fibula hypertrophy and remodeling. We did not directly compare VFG with or without allografts to other reconstruction options, so the decision to use this approach should be made thoughtfully and only after considering the potential serious risks.

**Level of Evidence:**

Level IV, therapeutic study.

## Introduction

The tibia is the second most-frequent site of primary bone tumors, after the femur [4, 5]. In most patients, the tumor extends in the metaepiphysis and is treated with an intra-articular resection with articular surface removal to obtain adequate surgical margins. Less frequently, when the tumor arises in the metadiaphysis, an intercalary resection is feasible, with native joint preservation [4, 5]. Patients with primary malignant bone tumors are often young with high functional demands, and their survival has been improved by applying appropriate chemotherapy protocols. Therefore, a durable reconstruction that lasts for many years is advised for these patients.

Several options to reconstruct large intercalary tibial defects have been reported, such as intercalary prostheses [13, 24, 26], massive allografts [2, 11, 23], bone transport [8], osteoinductive membrane techniques [25], recycled autografts [17, 19, 21, 32, 33], vascularized autografts [22], and a combination of massive allografts or cortical struts and vascularized autografts [1, 16, 18, 20].

Among vascularized autografts, the fibula is frequently used to restore large segmental bone defects, according to the length of the vascular pedicle and ease of access for harvest [7]. Intercalary vascularized fibula grafts (VFG) provide many advantages, such as early osteotomy union and spontaneous healing after fracture; they maintain the ability to hypertrophy under mechanical stress, even in critical soft tissue conditions [7]. Reconstruction with VFG was first reported by Taylor et al. [27], and Weiland et al. [31] described the first application after tumor resection. Capanna et al. [6] first described a VFG and massive allograft as a reconstruction option after intercalary resection for primary bone tumors; this technique is used because it combines the primary mechanical stability of the allograft and long-term biological potential of the vascularized fibula. Since then, several studies have reported results using VFG, either combined with a massive allograft or not, to reconstruct large bony defect of the tibia [1, 16, 18, 20, 22]. We believe it is important to report our results of VFG tibial reconstruction after intercalary resection for primary bone tumors in patients with a median of 12 years of follow-up. Because we have substantial experience using VFGs for intercalary defects, we thought it would be helpful to present our results, although we were not able to directly compare them with a comparison group.

We therefore asked: (1) What was the proportion of complications after reconstruction with VFG, with or without allografts? (2) What was the functional result after surgical treatment as assessed by the Musculoskeletal Tumor Society (MSTS) score? (3) What was the survivorship of these grafts free from revision and graft removal?

## Patients and Methods

### Study Design and Setting

This was a retrospective study performed during a 30-year period at the Careggi University Hospital of Florence by experienced orthopaedic oncology and plastic surgery teams.

### Patients

Between 1988 and 2021, 219 patients were treated at our institution for a primary malignant or aggressive benign bone tumor of the tibia with en bloc resection; we excluded patients treated with extra-articular resection or amputation. Of those, 54% (119 patients) had proximal tibial tumors with epiphyseal involvement and were treated with intra-articular resection and reconstruction with an osteoarticular allograft, allograft-prosthesis composite (APC), or modular prosthesis according to age, diagnosis, and preoperative or postoperative radiotherapy. Nine percent (20) of patients had distal tibial tumors that were treated with intra-articular resection and reconstruction with ankle arthrodesis using allogenic or autologous grafts, and 0.5% (1) underwent total tibial resection for extensive tumoral involvement of the tibia and reconstruction with an APC.

**Fig. 1:**
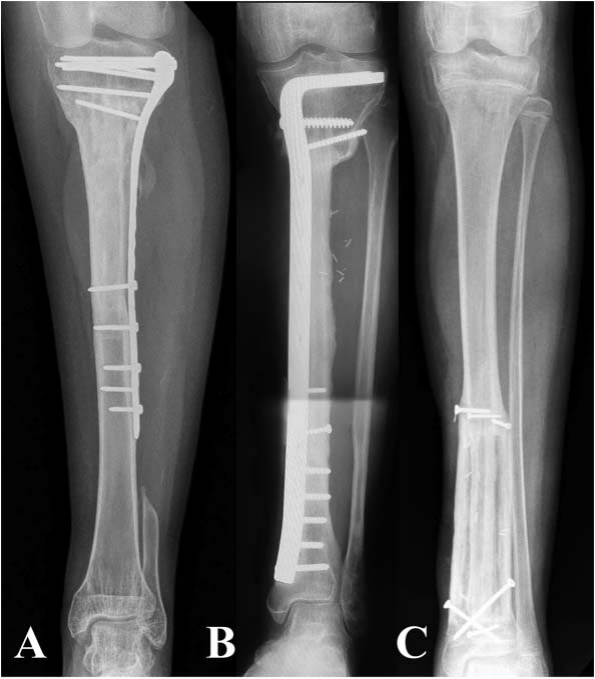
This figure shows the three types of VFG used in this study: (**A**) VFG combined with a concentric massive allograft, (**B**) VFG alone, and (**C**) VFG combined with allogenic cortical struts.

**Fig. 2:**
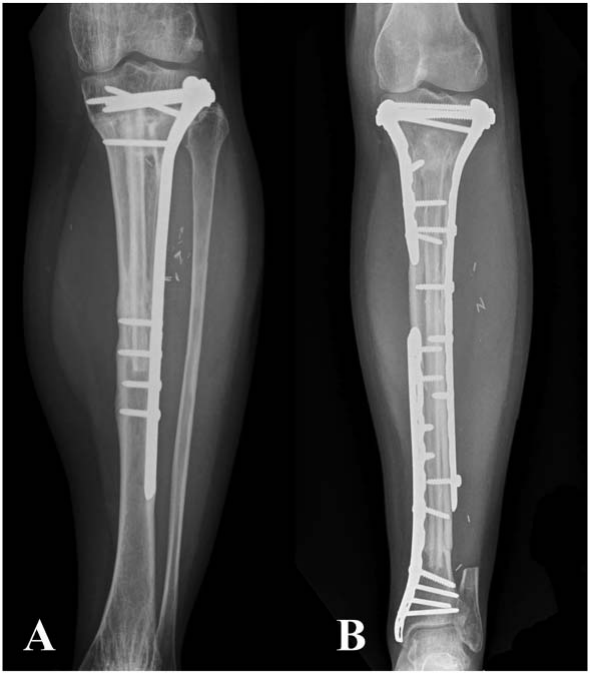
This figure shows the two types of bridging fixation: (**A**) fixation with a single bridging plate and (**B**) fixation with double plates (one bridging and two separate plates at osteotomy sites).

**Fig. 3:**
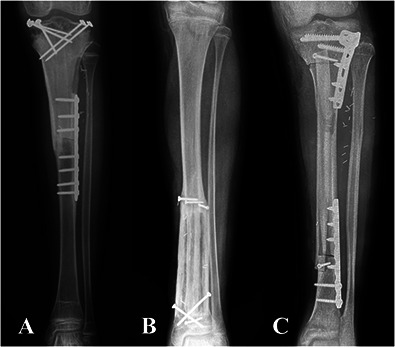
This figure shows the three types of nonbridging fixation: (**A**) fixation with proximal screws and a distal plate, (**B**) fixation with only screws or K-wires, and (**C**) fixation with double plates (one proximal and one distal).

**Fig. 4:**
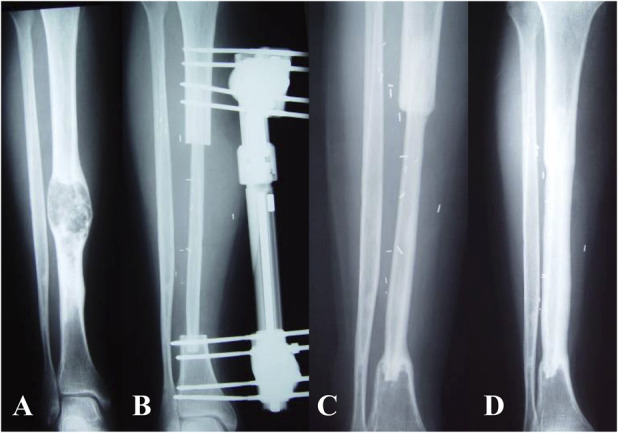
(**A**) This radiograph shows a diaphyseal osteoblastic osteosarcoma in a 19-year-old man. (**B**) The patient underwent intercalary resection of the tibia and reconstruction with VFG and external fixation. (**C**) After union of both osteotomies was achieved at 6 months after the index surgery, the external fixator was removed. (**D**) This radiograph shows progressive hypertrophy and remodeling of the vascularized fibula under mechanical stress once hardware was removed.

Thirty-six percent (79) of patients had metadiaphyseal bone tumors of the tibia and were treated with intercalary joint-sparing resection. We routinely use reconstruction with VFG after intercalary tibial resection for primary malignant or aggressive benign bone tumors in patients with long life expectancy and high functional demands and in whom at least 1 cm of residual bone stock of the proximal or distal epiphysis can be preserved. By contrast, we routinely use intercalary massive allograft reconstruction in short resections or in patients with metastatic disease who do not have long life expectancy. We avoid VFGs in patients with tibial bone metastasis, patients older than 70 years, or primary bone tumors in patients who may undergo postoperative radiotherapy; in these patients, we adopt alternative reconstructive methods such as intercalary prostheses, plate and cement, or intramedullary nailing with cement augmentation. According to the above-mentioned indications, 6% (5 of 79) of patients underwent massive allograft reconstruction because they were young and had intercalary resections shorter than 7 cm or metastatic disease at diagnosis without long life expectancy, whereas 94% (74 of 79) of patients underwent VFG reconstruction. VFG was used with massive allografts in 55 patients, alone in 12 patients, and combined with allogenic cortical bone struts in seven (Fig. 1). Our policy was to use VFG combined with a massive allograft in patients undergoing juxta-articular, joint-sparing resections that left less than 3 cm of residual epiphyseal bone, for intra-epiphyseal resections, or for long intercalary resections wherein the allograft can provide better mechanical stability. In these clinical situations, a VFG and massive allograft allow more-stable fixation and better tendinous reattachment of the patellar tendon. VFG was used with cortical bone struts in distal tibia intercalary resections where the narrow diameter of the allograft did not allow concentric assembling with the fibula. Finally, VFG alone was often used after mid- or distal tibia intercalary resection in patients with critical soft tissue conditions because of previous surgery, in whom the combination with a massive allograft would result in a bulkier reconstruction. Bone fixation was performed with a bridging plate in 31 patients, proximal screws and distal plate in 21, only screws or K-wires in eight, two plates (one proximal and one distal) in seven, temporary external fixation in four, and double plates (one bridging and two separate ones at the osteotomy site) in three. External fixation was removed once both osteotomies achieved union, generally after 6 months. A single-bridging or double-bridging plate was classified as bridging fixation (Fig. 2); proximal screws and distal plate, screws or K-wires only, and two separate plates were considered nonbridging fixation (Fig. 3). Temporary external fixation was considered a different type of fixation (Fig. 4).

All 74 patients who were treated with VFGs were available at a minimum follow-up of 2 years, with a median follow-up duration of 12.3 years (range 2 to 35 years). Nine patients have not been seen in the past 5 years and are not known to have died; however, they had 3.4, 3.8, 3.8, 7.7, 9.7, 10, 10, 11.4, and 13.8 years of follow-up, and they were included.

### Descriptive Data

The median age at the time of operation was 16 years (range 5 to 68 years); 46% (34) of patients were women and 54% (40) were men. Furthermore, 53% (39) were skeletally immature patients, while 47% (35) were skeletally mature. The median tibial resection length was 15 cm (range 7 to 27 cm), leaving a median proximal residual tibial bone of 3 cm (range 1 to 18 cm) and a distal residual tibial segment of 15 cm (range 7 to 27 cm); the mean fibular resection length was 18 cm (range 10 to 29 cm) (Table 1). All patients had malignant bone tumors or locally aggressive, benign tumors. The diagnoses were high-grade osteosarcoma (22), Ewing sarcoma (19), adamantinoma (16), low-grade osteosarcoma (5), fibrosarcoma (3), malignant fibrous histiocytoma and Grade 2 chondrosarcoma (2), malignant myoepithelioma (1), angiosarcoma of bone (1), malignant peripheral nerve sheath tumor of bone (1), squamous cell carcinoma secondary to chronic osteomyelitis (1), and desmoplastic fibroma (1). Experienced pathologists made the diagnosis after biopsy in all patients. At diagnosis, five patients had a pathologic fracture and, according to the MSTS staging system [9], one benign tumor was Stage 2, three malignant tumors were Stage IA, 20 were IB, two were IIA, 47 were IIB, and one was Stage IIIB.

**Table 1.** Patient characteristics

Characteristic	Total (n = 74)
Women	46% (34)
Age in years	16 (5-68)
Diagnosis	
Osteosarcoma (high-grade)	30% (22)
Ewing sarcoma	26% (19)
Adamantinoma	22% (16)
Osteosarcoma (low-grade)	7% (5)
Fibrosarcoma	4% (3)
Chondrosarcoma (Grade 2)	3% (2)
Malignant fibrous histiocytoma	3% (2)
Malignant myoepithelioma	1% (1)
Angiosarcoma of bone	1% (1)
MPNST	1% (1)
Squamous cell carcinoma secondary to chronic osteomyelitis	1% (1)
Desmoplastic fibroma	1% (1)
Stage	
2	1% (1)
IA	4% (3)
IB	27% (20)
IIA	3% (2)
IIB	64% (47)
IIIB	1% (1)
Treated with chemotherapy	57% (42)
Treated with radiotherapy	0% (0)
Tibial resection length in cm	15 (7-27)
Intercalary	76% (56)
Intraepiphyseal	24% (18)
Proximal residual juxta-articular bone	3 (1-18)
Distal residual juxta-articular bone	15 (2-24)
Fibular resection length in cm	18 (10-29)
Free VFG	91% (67)
Pedicle VFG	9% (7)
Reconstruction	
VFG + concentric massive allograft	74% (55)
VFG	16% (12)
VFG + allogenic cortical struts	10% (7)
Fixation	
Single bridging plate	42% (31)
Double bridging plate	4% (3)
Proximal screws + distal plate	28% (21)
Only screws/K-wires	11% (8)
Proximal and distal plates	10% (7)
External fixation	5% (4)

Data presented as % (n) or median (range). MPNST = malignant peripheral nerve stealth tumor; VFG = vascularized fibula graft.

### Surgical Technique, Aftercare, and Cancer Treatment

All operations were performed with the patient in the supine position, through an extended anteromedial or anterolateral approach according to the tumor extension. Eighteen patients underwent intraepiphyseal resections. A histologic examination of the resected tumors found that surgical margins were wide in 69 patients, marginal in four, and wide-contaminated in one.

The vascularized fibula was harvested from the contralateral leg in 67 patients, whereas an ipsilateral pedicle fibula was used in seven. The free VFG was harvested by a microsurgical team using separate instruments, who took care to avoid contamination between the two surgical fields through a posterolateral approach. The pedicle VFG was harvested through a posterolateral approach, with a double incision on the same leg. The median length of the fibular graft was 18 cm (range 10 to 29 cm). The harvested fibula was at least 2 cm longer than the tibial resection length to obtain a minimum overlap of 1 cm for each osteotomy. Primary syndesmotic screw fixation was performed at the ankle of the donor side in two children; we routinely do not use primary syndesmotic fixation in patients with more than 7 cm of residual distal fibula. In patients with free VFG reconstruction, the vascular pedicle of the flap, including the peroneal artery and two vena comitans, was anastomosed in 64 patients with the collateral branch of the anterior tibial artery and vein, while in two and one patient, it was anastomosed with the collateral branch of the posterior tibial and peroneal vessels, respectively. Moreover, in eight patients, the free VFG was harvested with the fasciocutaneous flap based on its perforator vessels, and in one patient, it was harvested with the soleus muscle flap to cover skin defects secondary to tumor excision.

The median operative time was 8 hours (range 4 to 12 hours); in patients with free VFG reconstruction, the median operative time was 8 hours (range 4 to 12 hours), and in patients with pedicle VFG, it was 7 hours (range 5 to 9 hours).

All patients received perioperative antibiotic prophylaxis with intravenous vancomycin (1 g every 12 hours) combined with tobramicin (1 mg/kg every 8 hours) until 2010 and with piperacillin and tazobactam (4.5 g every 8 hours) from 2010 onward, following our institution's protocol; this was continued until drain removal. Postoperatively, in patients in whom the patellar tendon was reattached and in pediatric patients, the operated-on leg was protected with a long leg cast for 4 weeks. Controlled passive movements of the knee and ankle were then allowed. In young and adult patients with intercalary resections distal to the tibial apophysis, controlled passive ankle and knee movement was allowed postoperatively. No weightbearing was allowed until radiographic evidence of VFG union, which was considered as the disappearance of the fibula’s osteotomy line or the appearance of a bridging callus on radiographs in patients with VFG and allograft or VFG alone. Then, the patient could walk with partial weightbearing, and full weightbearing was started after evidence of complete allograft union. The donor leg was left free postoperatively, encouraging active and passive movements of the knee, ankle, and toes. Full weightbearing on the donor side was allowed 3 weeks after surgery.

Forty-one patients with a diagnosis of high-grade osteosarcoma, Ewing sarcoma, malignant myoepitelioma, high-grade fibrosarcoma, or malignant fibrous histiocytoma received preoperative and postoperative chemotherapy, whereas one 67-year-old man with a diagnosis of high-grade osteosarcoma underwent postoperative chemotherapy only. No patients underwent radiation therapy. In patients treated with preoperative chemotherapy, the surgery was usually planned 3 weeks after the last drug administration, which is not different from other reconstructive options.

### Data Sources and Variables

All patients were periodically reviewed according to oncologic follow-up, and they underwent clinical and radiologic examinations. Postoperatively, we followed patients with malignancies every 3 months during the first 2 years, every 4 months during the third year, every 6 months during the fourth and fifth years, and yearly until the 10th year after the index surgery. Data extracted from medical records were registered in our database.

At the most-recent clinical follow-up, at a median of 12.3 months (range 2 to 35), 72% (53 of 74) of patients were continuously disease-free. Ten patients had no evidence of disease after treatment of local recurrence (five patients) and distant metastasis (five). One patient with Ewing sarcoma developed bone metastasis 2.3 years after the index surgery and is alive with disease at 6.4 years. Nine patients died of disease after a median of 5 years from the index surgery (range 2.3 to 11 years) because of metastatic disease, and one patient died because of another cause after 11.8 years.

### Primary and Secondary Study Outcomes

Our primary study endpoint was complications after surgery, which we assessed by chart review. Our secondary study endpoints were the MSTS score and survivorship. The MSTS score is an established system to assess functional outcomes in patients treated for bone tumors. In lower limb reconstructions, it evaluates six parameters: pain, function, emotional acceptance, support, walking, and gait, giving a value ranging from 0 to 5 according to specific criteria. The sum of the individual scores defines the overall functional score, with a maximum of 30 points [10]. The MSTS score was assessed in patients in whom the VFG was retained at the final clinical visit. Furthermore, the function of the knee and ankle was clinically assessed by clinicians using a goniometer for all patients, except for those who died or were lost to follow-up, in whom functional results were extracted through a clinical chart review; either MSTS score and knee and ankle ROM was assessed by independent observers and not by the surgeons who performed the procedures. We evaluated survivorship free from revision and survivorship free from graft removal using the Kaplan-Meier survivorship estimator.

In all patients, union and hypertrophy of the VFG were radiographically assessed; nonunion was defined as the lack of evidence of osteotomy healing on radiographs 9 months after the index surgery, and complications and graft removal were recorded during follow-up. We do not routinely monitor fibular vitality, such as VFG with a skin flap, based on perforator vessels, bone scan, or single-photon emission CT. We evaluated graft union and hypertrophy as signs of persistent vascular supply. We defined union on radiographs as cortical fusion of the allograft and VFG on AP and lateral views, and we defined hypertrophy as increase of the VFG in width, although we did not measure it in detail. Simultaneously, donor site morbidity at the final clinical visit was evaluated.

### Ethical Approval

Ethical approval for this retrospective observational study was obtained from our local institutional review board (ref. 10197/2017).

### Statistical Analysis

Reconstruction survival was determined according to the Kaplan-Meier method, with revision surgery for any complications and removal of the VFG as endpoints. Kaplan-Meier curves and survival proportions were computed using R version 4.1.2 via Survival version 3.5. In our study, however, the occurrence of death as a competing event was observed in a low proportion of patients, and only occurred after the primary event of interest had already occurred. Considering the nature of our data, we did not consider death after the primary event of interest as a competing event. A log-rank test was used to compare the survival distributions. Significance was set at p < 0.05.

## Results

### Complications and Reoperations

Donor site complications were observed in 14% (10 of 74) of patients. Seven percent (5) of patients experienced a mild ankle valgus deformity 7 to 10 years after the index surgery and all of these patients were treated nonoperatively. Five patients had claw toes that involved the first toe in four patients, all treated nonoperatively. In the fifth patient, the other toes were involved and managed with surgical tendon release. During follow-up, 62 complications were observed at the recipient site in 64% (47) of patients at a median of 11 months (range 0 to 237) after the primary procedure. Surgical revision of these complications was performed in 49% (36) of patients after a median of 12 months (range 0 to 237), and VFG removal was performed in 11% (8) after a median of 12 months (range 5 to 94) (Table 2).

**Table 2.** Recipient-site complications that underwent surgical treatment

Patient	Reconstruction type	Fixation type	Complication type	Follow-up, months	Treatment of complication	Outcome	VFG removal (months)	MSTS score
1	VFG + Allograft	1	Distal tibia skip metastasis	45	New distal tibial intercalary resection preserving the previous free VFG and ipsilateral VFG reconstruction	NED	No	30
2	VFG + Allograft	1	Deep infection	2	Debridement and local flap covering and antibiotics	Healed	No	28
3	VFG + Allograft	1	Nonunion	10	Autologous graft augmentation and new synthesis	Healed	No	30
4	VFG + Allograft	1	Wound dehiscence	1	Debridement	Healed	No	30
			Achilles tendon retraction	30	Tendon elongation	Resolved	No	
5	VFG + Allograft	1	Nounion	12	Autologous graft augmentation and new synthesis	Healed	No	26
6	VFG + Allograft	1	Nounion	9	Autologous graft augmentation	Healed	No	30
7	VFG + Struts	1	Nonunion	36	Autologous graft augmentation and new synthesis	Healed	No	28
8	VFG + Allograft	1	LR	7	Above-knee amputation	DOD	Yes (7)	-
9	VFG + Allograft	2	Fracture	64	Autologous graft augmentation and new synthesis	Healed	No	30
10	VFG alone	1	Nonunion with plate rupture	28	Autologous graft augmentation and new synthesis	Healed	No	25
11	VFG + Allograft	2	Fracture	24	New synthesis with bridging fixation	Healed	No	30
12	VFG + Allograft	2	Screw-related pain	72	Screw removal	Resolved	No	30
13	VFG + Allograft	1	Deep infection	19	Debridement and antibiotics	Resolved	No	25
			Nonunion	47	Autologous graft augmentation and new synthesis	Healed	No	
14	VFG + Struts	2	Wound dehiscence	1	Debridement and local flap cover	Healed	No	30
15	VFG + Allograft	1	Proximal tibia varus deformity	85	Deformity correction	Resolved	No	24
			Traumatic tibial apophysis detachment	89	ORIF	Healed	No	
16	VFG + Allograft	2	Fracture	34	Autologous graft augmentation and new synthesis	Healed	No	24
			Common peroneal nerve palsy	50	Tibialis posterior proanterior tendon trasposition	Resolved	No	
17	VFG + Allograft	2	Wound dehiscence	1	Debridement	Healed	No	28
18	VFG + Allograft	2	Fracture	40	Autologous graft augmentation and new synthesis	Healed	No	28
19	VFG + Allograft	2	Nonunion	10	Autologous graft augmentation and new synthesis	Healed	No	29
20	VFG + Allograft	1	Soft tissue LR	39	Excision	NED	No	30
21	VFG + Allograft	2	Nonunion	9	Autologous graft augmentation and new synthesis	Healed	No	-
			Soft tissue LR	75	Excision	DOD	No	
22	VFG alone	1	Deep infection	18	Debridement, plate removal, and antibiotics	Healed	No	26
23	VFG + Allograft	1	Osteoarthritis	237	TKA	Resolved	No	30
24	VFG + Allograft	2	Fracture	13	Autologous graft augmentation and new synthesis	Fracture nonunion	No	-
			Bone LR	25	Above-knee amputation	DOD	Yes (25)	
25	VFG + Allograft	2	Wound dehiscence	1	Debridement and local flap cover	Healed	No	30
26	VFG + Allograft	2	Necrosis of the skin island	0	Debridement and local flap cover	Healed	No	30
			Nonunion	37	Autologous graft augmentation and new synthesis	Healed	No	
			6 cm hypometria	62	Elongation with Ilizarov frame	Resolved		
27	VFG + Allograft	2	Bone LR	5	Above-knee amputation	NED	Yes (5)	-
28	VFG + Allograft	2	Fracture	75	ORIF with new synthesis	Nonhealing with nonviable VF, VFG removal and allograft reconstruction	Yes (94)	-
29	VFG + Allograft	2	Deep infection	4	Debridement, local flap coverage, and antibiotics	Persistent infection, above-knee amputation with infection resolution	Yes (11)	-
30	VFG alone	1	Plate-related pain	13	Plate removal	Resolved	No	30
			Bone LR	177	Intercalary resection and reconstruction with induced-membrane technique	NED	No	
31	VFG + Allograft	1	VFG vein thrombosis	0	New anastomoses with safena vein graft	Resolved	No	30
32	VFG + Allograft	1	Nonunion with plate rupture	6	Autologous graft augmentation and new synthesis	Healed with residual valgus deformity	No	23
33	VFG alone	1	Bone LR	12	Below-knee amputation	NED	Yes (12)	-
34	VFG + Allograft	2	Soft tissue LR	28	Excision	DOD	No	28
35	VFG alone	2	Deep infection	4	Debridement, local flap covering + antibiotics	Persistent infection. Above knee amputation	Yes (26)	-
36	VFG alone	2	Deep infection	1	Debridement and antibiotics	Persistent infection. Nonviable VFG removal and reconstruction with bone transport. Healed	Yes (12)	-

ORIF = open reduction and internal fixation; VFG = vascularized fibula graft; MSTS = Musculoskeletal Tumor Society; LR = local recurrence; 1 = bridging fixation; 2 = nonbridging fixation; 3 = external fixation; NED = no evidence of disease; DOD = dead of disease.

Five patients had postoperative common peroneal nerve palsy that completely recovered spontaneously in four patients; four patients developed wound dehiscence, while one patient had necrosis of the skin island and another had venous thrombosis of the VFG pedicle within the first hours postoperatively. There were 12 nonunions in 10 patients (14%), with hardware breakage in two. These patients were treated with graft augmentation with autologous bone of the iliac crest and new fixation in all patients except one, in whom the original fixation was retained. Twenty-two percent (16 of 74) had a fracture at a median of 24 months (range 4 to 75) after the primary surgery that healed spontaneously with brace protection in 10 patients, whereas six patients had surgical revision with a new bridging plate fixation. One of them underwent removal of a nonviable VFG and new reconstruction with a massive allograft and new plate fixation. With the numbers we had, the only factor associated with the risk of fracture was fixation with a temporary external fixator (Table 3). Deep infection was observed in 8% (6) of patients at the recipient site at a median of 4 months (range 1 to 19) after the primary surgery (Table 2), leading to VFG removal in three patients. No patient had an infection at the donor site. Twelve percent (9) of patients had local recurrence at a mean of 46 months (range 5 to 177) after tumor excision, leading to amputation in four patients (Table 2). The risk of local recurrence was higher in patients with distal tibial intercalary resections (hazard ratio 0.71 [95% confidence interval 0.57 to 0.9]; p = 0.03).

**Table 3.** Recipient-site mechanical and infective complications

Variable (total)	Nonunion (10)			Fracture (16)			Infection (6)		
	% (n)	HR (95% CI)	p value	% (n)	HR (95% CI)	p value	% (n)	HR (95% CI)	p value
Sex Female (34) Male (40)	9 (3)	2.3 (0.61-8.6)	0.22	15 (5)	1.4 (0.52-3.9)	0.49	9 (3)	1.3 (0.21-7.5)	0.81
	18 (7)			28 (11)			8 (3)		
Resection type Intercalary (56) Intra-epiphyseal (18)	16 (9)	0.27 (0.04-2.1)	0.21	18 (10)	1.9 (0.68-5.1)	0.23	9 (5)	0.61 (0.072-5.2)	0.66
	6 (1)			33 (6)			6 (1)		
VFG harvest Free (67) Ipsilateral pedicle (7)	13 (9)	1.2 (0.16-9.8)	0.83	22 (15)	0.79 (0.1-6)	0.82	6 (4)	3.6 (0.64-20)	0.15
	14 (1)			14 (1)			29 (2)		
Reconstruction type VFG alone (12) VFG + allograft (55) VFG + struts (7)	8 (1)	Reference		33 (4)	Reference		25 (3)		
	15 (8)	0.89 (0.19-4.2)	0.88	18 (10)	0.52 (0.16-1.7)	0.27	6 (3)	0.23 (0.05-1.2)	0.08
	14 (1)	0.80 (0.07-8.8)	0.86	29 (2)	0.91 (0.16-5.0)	0.91	0 (0)	NA	1
Fixation type Bridging fixation (34) Non-bridging fixation (36) External fixation (4)	21 (7)	Reference		9 (3)	Reference		9 (3)		
	8 (3)	0.3 (0.08-1.2)	0.09	28 (10)	2.1 (0.7-6.9)	0.21	8 (3)	0.97 (0.19-4.8)	0.97
	0 (0)	NA	1	75 (3)	9.6 (2.3-45.8)	0.03	0 (0)	NA	1
Chemotherapy No (32) Yes (42)	6 (2)	1.9 (0.5-7.1)	0.35	13 (4)	2.1 (0.66-6.6)	0.21	9 (3)	0.66 (0.13-3.3)	0.62
	19 (8)			29 (12)			7 (3)		
Growth Skeletally immature (39) Skeletally mature (35)	15 (6)	0.65 (0.19-2.2)	0.49	28 (11)	0.48 (0.17-1.4)	0.17	10 (4)	0.55 (0.1-3)	0.49
	11 (4)			14 (5)			6 (2)		

Overall, 8% (6 of 74) of patients underwent amputation because of infectious complications (2 of 6) and local recurrence (4 of 6).

At the final clinical visit, a mean limb-length discrepancy of 2.4 cm (range 1 to 6) was observed in 35% (26) of patients and in 83% (15 of 18) of skeletally immature patients treated with intra-epiphyseal resections. A shoe lift was used in all patients except one, a 10-year-old child treated for a high-grade osteosarcoma with an intraepiphyseal resection; this child had a 6-cm limb length discrepancy that was managed with bone transport of the femur using external fixation 62 months after the index procedure. Finally, one patient developed a postoperative deep vein thrombosis that was treated with lifelong oral anticoagulant therapy.

### MSTS Scores and Knee Function

At the final follow-up interval, after a median of 12.3 years (range 2 to 35), the median MSTS score was 30 (range 23 to 30). The MSTS score was assessed only in patients in whom the VFG was retained at the final clinical visit; if we had included patients who had an amputation, the MSTS scores would have been lower. Pain and emotional acceptance scores ranged between 4 and 5 points, and function, support, walking, and gait scores ranged between 3 and 5 points. Regarding knee function, the median active flexion was 140° (range 100° to 140°), and an extension lag ranging between 5° and 20° was found in eight patients. For ankle function, the median active flexion was 50° (range 10° to 50°) and median active extension was 20° (range 5° to 20°).

### Survivorship of VFGs

**Fig. 5:**
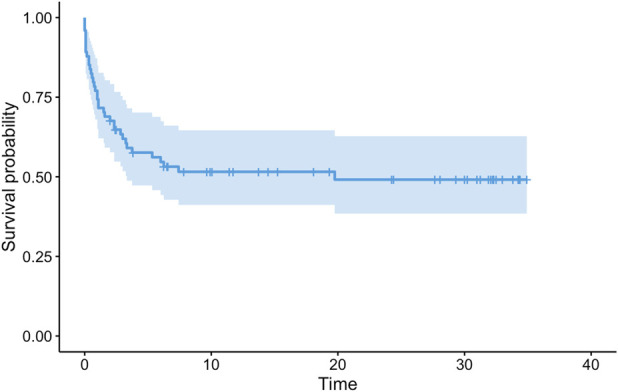
This graph shows revision-free survival, with surgical revision for any complication as the endpoint. The survival was 58% (95% CI 47% to 70%) at 5 years, 52% (95% CI 41% to 65%) at 10 and 15 years, and 49% (95% CI 38% to 63%) at 20 and 30 years. The light blue area represents CIs.

**Fig. 6:**
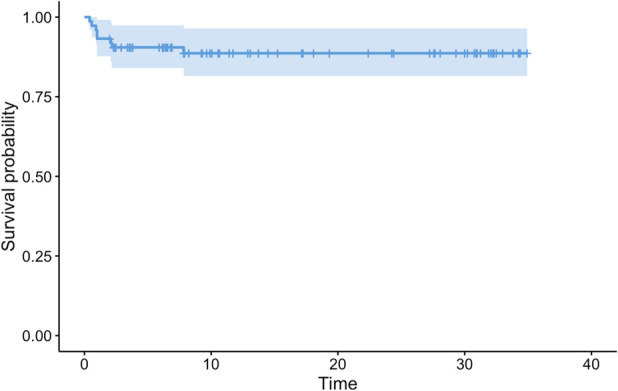
This graph shows VFG removal-free survival, with graft removal for any complication as the endpoint. The survival was 91% (95% CI 84% to 98%) at 5 years and 89% (95% CI 82% to 97%) at 10 to 30 years. The light blue area represents CIs.

The revision-free survival of reconstructions, with revision surgery for any complication as the endpoint, was 58% (95% CI 47% to 70%) at 5 years, 52% (95% CI 41% to 65%) at 10 and 15 years, and 49% (95% CI 38% to 63%) at 20 and 30 years (Fig. 5). Except for five instances, all complications that led to surgical revision occurred during the first 5 years postoperatively. The overall reconstruction survival, with VFG removal or amputation as the failure endpoint, was 91% (95% CI 84% to 98%) at 5 years and 89% (95% CI 82% to 97%) at 10 to 30 years (Fig. 6). Only 4 of 8 patients underwent VFG removal because of mechanical or infective complications.

## Discussion

After intercalary tibial resection, the objective of reconstruction is to restore lower limb function with a low risk of reoperation and implant removal in the long term, while considering the young age and high functional expectations of patients with primary bone tumors. Autologous VFG, either pedicled from the ipsilateral leg or a free flap harvested from the contralateral leg, is a possible reconstructive method. After intercalary tibial resection, VFG can be used alone or combined with allogenic cortical struts or a massive allograft. In the present study, we aimed to investigate the results of this technique, which we anticipated would result in a functional, durable, joint-sparing, biological reconstruction and avoid a large metallic endoprosthesis. We found that although the MSTS function score was good for those who maintained their reconstruction at the final follow-up, approximately half of patients had one or more complication and nearly 10% of patients ultimately underwent amputation (and were not counted in the functional analysis). Despite a consistent risk of mechanical complications in the first 5 years postoperatively, with complication-free survival of nearly 60% at 5 years, almost 90% of the patients retained their VFG at the most recent follow-up. The proportion of patients sustaining fractures may be lower with long-spanning plate fixation, and nonbridging fixation or temporary external fixation helps to enhance fusion and hypertrophy of the fibula in young patients, but we could not confirm that in this study.

### Limitations

First, there might have been selection bias regarding the indication for intercalary joint-sparing resection and VFG reconstruction rather than intra-articular resection and prosthetic reconstruction, but when feasible, we aim to preserve native joints in all patients with primary bone tumors. Second, nine patients were not seen in the past 5 years, and they could have been treated for other complications elsewhere. However, all nine patients had long-term follow-up confirming functional and survival results. Third, we did not analyze a control group with alternative osteoarticular reconstruction, instead focusing on joint-sparing resection. In our opinion and experience, we believe the functional advantages of preserving the native joints and their tendinous insertions are substantial. Fourth, we did not analyze a control group with alternative intercalary reconstruction, such as prostheses, massive allografts, or recycled autografts, but we aimed to investigate the incidence and type of complications and survival of a vascularized reconstruction, with or without allograft augmentation. Fifth, VFG reconstructions with and without massive allografts or allogenic struts were included, but we analyzed the incidence of complications according to different reconstructive options, and the numbers are too small to detect a difference in complication risk between these reconstructive types. Sixth, both pedicle and free VFG reconstruction were included, with possible bias because of different complication risks, but in our experience, the proportion of complications was similar to that reported by other authors [18]. Seventh, we did not have sufficient numbers to compare our results by gender. Men seemed to have a higher proportion of mechanical complications, but with the numbers we had, this difference was not significant (Table 3). A larger study is necessary to address this issue. Finally, we did not consider the function of patients who underwent amputation when we calculated the MSTS function score. If we had included these patients, the score would likely have been lower. We aimed to show the function of patients who retained their VFG, but this score does not apply to all patients at diagnosis.

### Complications and Reoperations

In our series, 49% of patients underwent surgical revision for complications, but most complications occurred within the first 5 years postoperatively, and all but eight patients retained their VFG at the final clinical follow-up. Nonunions and fractures were the most frequent complications in our study. No differences in nonunion were seen between VFG alone and VFG combined with cortical struts or massive allografts, but we likely did not have sufficient numbers of patients with each technique to address this. However, nonunion was more frequent in patients with bridging plate fixation, but this difference did not reach statistical significance. One might assume there is a higher risk of nonunion in spanning fixation related to stress shielding induced by the bridging plate, which could inhibit fibular hypertrophy, but we did not have sufficient numbers to address this. In our experience, the nonunion risk did not seem to be related to resection length, proximal and distal residual bone, gender, patient age, or chemotherapy, as reported [15]. All patients who experienced nonunions healed after augmentation with iliac crest autologous bone grafts and new fixation.

**Fig. 7:**
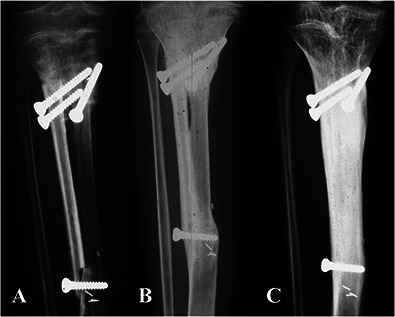
(**A**) This radiograph is from an 11-year-old girl treated for an osteoblastic osteosarcoma of the tibia with intercalary resection and reconstruction with VFG combined with an allogenic fibular graft and screws-only fixation. Radiographs show (**B**) progressive hypertrophy of the fibula 2 years after the index surgery and (**C**) abundant hypertrophy, remodeling, and fusion of the VFG with the cortical strut 5 years after the primary surgery.

Fractures were observed in 22% of patients, all of which healed after internal fixation or nonoperative treatment, except for one patient who underwent removal of a nonviable VFG and new reconstruction with an intercalary massive allograft. The proportion of patients with fractures appeared higher after removal of external fixators; furthermore, fractures were more frequent in patients with nonspanning fixation, but with the numbers of patients we had, we could not confirm this. Indeed, one drawback of VFG intercalary reconstruction in the lower limb is the low mechanical strength of the fibula. To improve mechanical strength, combining a VFG with long bridging plate fixation or a massive allograft has been suggested [28, 30]. We observed no difference in the proportion of fractures between VGF alone and VFG combined with an allograft or allogenic struts. Again, this may be related to small numbers or perhaps the fixation type is an important factor in preventing fractures. Future studies are needed to address this. There may be a higher incidence of fractures in growing patients, but again, we could not substantiate that it was higher, and if it is, it may be related to the higher number of nonspanning fixations in this age group. Based on our experience, we believe that when feasible, long-spanning plate fixation may be preferable in both types of reconstruction. Furthermore, in a very long intercalary resection, long-bridging plate fixation with a second plate for each osteotomy may be helpful, as reported [3]. Conversely, in joint-sparing, intraepiphyseal resection of the proximal tibia that preserves a very short segment of residual bone, we believe reconstruction with VFG combined with a massive allograft is preferred, and nonspanning fixation with proximal screws and distal plate is acceptable if there is no space for a bridging plate. We currently use a VFG and massive allograft to improve mechanical stability and obtain more-stable fixation in joint-sparing resection when a short proximal residual bone does not allow for stable spanning plate fixation. Furthermore, in very young patients or in patients with critical soft tissue conditions, nonbridging fixation (Fig. 7) or temporary external fixation (Fig. 4) may be preferred to obtain appropriate fibular hypertrophy and remodeling, despite the higher risk of fracture. Unfortunately, with the numbers we had, we could not definitively document the superiority of one approach over another.

In our series, the only factor that might be associated with infection was reconstruction with an ipsilateral VFG, although we could not demonstrate a difference with the numbers of patients we had. Other authors [18] have reported a shorter operative duration with a similar complication rate using an ipsilateral or free VFG. Because of the low number of patients treated with this technique compared with free VFGs, and despite the double incision used to excise the tumor and harvest the fibula (which could increase infections), we could not show a difference. We now routinely prefer to harvest the VFG from the contralateral limb to avoid a double incision on the same leg and prevent excessive weakening of the operated-on leg and instability of reconstruction related to the lack of the fibula, although we did not assess whether there was a difference in mechanical complications between these two groups. Further studies comparing the long-term failure rates of free and pedicle VFG reconstruction should be performed.

The choice of a free VFG to reconstruct intercalary defects of the tibia has some drawbacks. One is donor site morbidity, which in our series was 14%, although it did not affect the patients’ quality of life. Another disadvantage is the long surgical time associated with the complexity of VFG harvesting and anastomosis, which can be reduced through the simultaneous work of orthopaedic and microsurgical teams. We did not have a comparison group to demonstrate how patients treated with this technique might compare with those with allografts alone.

### MSTS Scores and Knee Function

The median MSTS score was good, with excellent active ROM of the knee and ankle. However, this does not consider patients who had an amputation, so if these patients had been considered, the scores would certainly be lower. Such excellent functional results were probably related to retention of the native knees and ankles and their tendinous and ligament insertions. Joint-sparing intercalary tibial reconstructions have been reported to be successful [1, 13, 16-22, 32, 33]. By contrast, the functional outcomes of proximal tibial endoprosthetic reconstructions have been reported to be lower, with a mean MSTS of about 80% (40% to 90%), in the long term [12, 29].

### Survivorship of VFGs

The revision-free survival was almost 60% at 5 years and 50% at 20 and 30 years, and the overall survival of the reconstructions free from removal of VFGs was about 90% at 10 to 30 years. If we excluded patients treated for local relapse, 95% retained their reconstruction at the final clinical follow-up visit. In comparing our results with the reports of others, intercalary tibial prostheses are associated with a rate of loosening reaching 36% in some series with a mean follow-up of about 5 to 6 years [13, 24, 26], whereas intercalary massive allografts have been associated with an incidence of fractures and deep infections ranging between 38% and 62% [2, 11, 23]. Again, we cannot make a direct comparison in our study, and conditions probably differ among the studies. Reconstructions with intercalary recycled autografts, both frozen and irradiated, were characterized by an incidence of complications (between 18% and 50%) [21, 32, 33], although a major drawback of this technique is the absence of material for a histologic examination of chemotherapy-induced necrosis and surgical margins [32, 33]. An interesting reconstructive option combines a VFG and recycled autograft, with the potential advantages of early bone union and lower complication risk [17, 19]. Proximal tibia prosthetic reconstructions are also associated with complications that are possibly higher than we found with intercalary reconstruction, particularly for infections [12, 14, 29]. However, because these are much different procedures for possibly different indications and we did not directly compare patients with VFG to a similar group of patients treated with prosthetic reconstructions, we cannot confirm there is a difference in complications. Intercalary metal prostheses and massive allograft (without an associated vascularized fibula) have an implant removal-free survival of 63% at 10 years and 74% at 10 and 20 years [2, 24]. Furthermore, the incidence of mechanical failure does not appear to reach a definite plateau [23]. Proximal tibia prosthetic reconstructions have an implant survival rate ranging between 10% and 25% at 20 years, with a risk of deep infection that persists throughout the life of the prosthesis at a mean of 1% per year [12, 14, 29]. These reports may be less favorable than our outcomes regarding VFG reconstructions, but these reports cannot be directly compared with ours because of differences in indications, patient populations, and other factors. However, we believe that augmenting an intercalary massive allograft with a VFG to combine the early mechanical strength of the allograft with the biological potential of the vascularized fibula seems to result in a durable reconstruction and can preserve the native joint. Future research should compare long-term, failure-free survival of biologic and prosthetic joint-sparing tibial reconstructions.

### Conclusion

Reconstruction of intercalary tibial defects with VFG is a challenging option that necessitates a well-trained microsurgical team, but we believe the biological potential of a vascularized fibula is a major advantage. We also caution that this was associated with substantial complications in about half of our patients. Most complications were remedied with secondary procedures that retained the graft. In our experience, 90% of patients retained their graft at the final clinical follow-up; these patients had good function. However almost 10% had amputations, which reduces overall functional scores. Most failures were fractures and nonunions that occurred within the first years postoperatively. Once graft union and hypertrophy were achieved, the proportions of complications were low, confirming that biologic reconstruction with a viable graft can be a lasting solution after intercalary tibial resections. We believe that VFG represents an effective reconstruction option after tibial intercalary resection for primary bone tumors, and it is one alternative in active patients with long life expectancy. Larger studies comparing VFG with other reconstruction options such as allografts alone, intercalary endoprostheses, and recycled autografts are warranted to assess the potential benefits of one technique over the other. We believe that VFG, alone or combined with an allograft, is an effective method to reconstruct a functional lower extremity after intercalary resections of the tibia for primary bone tumors, providing a potentially durable reconstruction with excellent functional results.
